# Supplementary figures and images for: The immune receptor SLAMF5 regulates myeloid-cell mediated neuroinflammation in multiple sclerosis
Source: PLoS Biol. 2025 Sep 8;23(9):e3003373. doi: 10.1371/journal.pbio.3003373 (PMC12431667; doi:10.1371/journal.pbio.3003373)

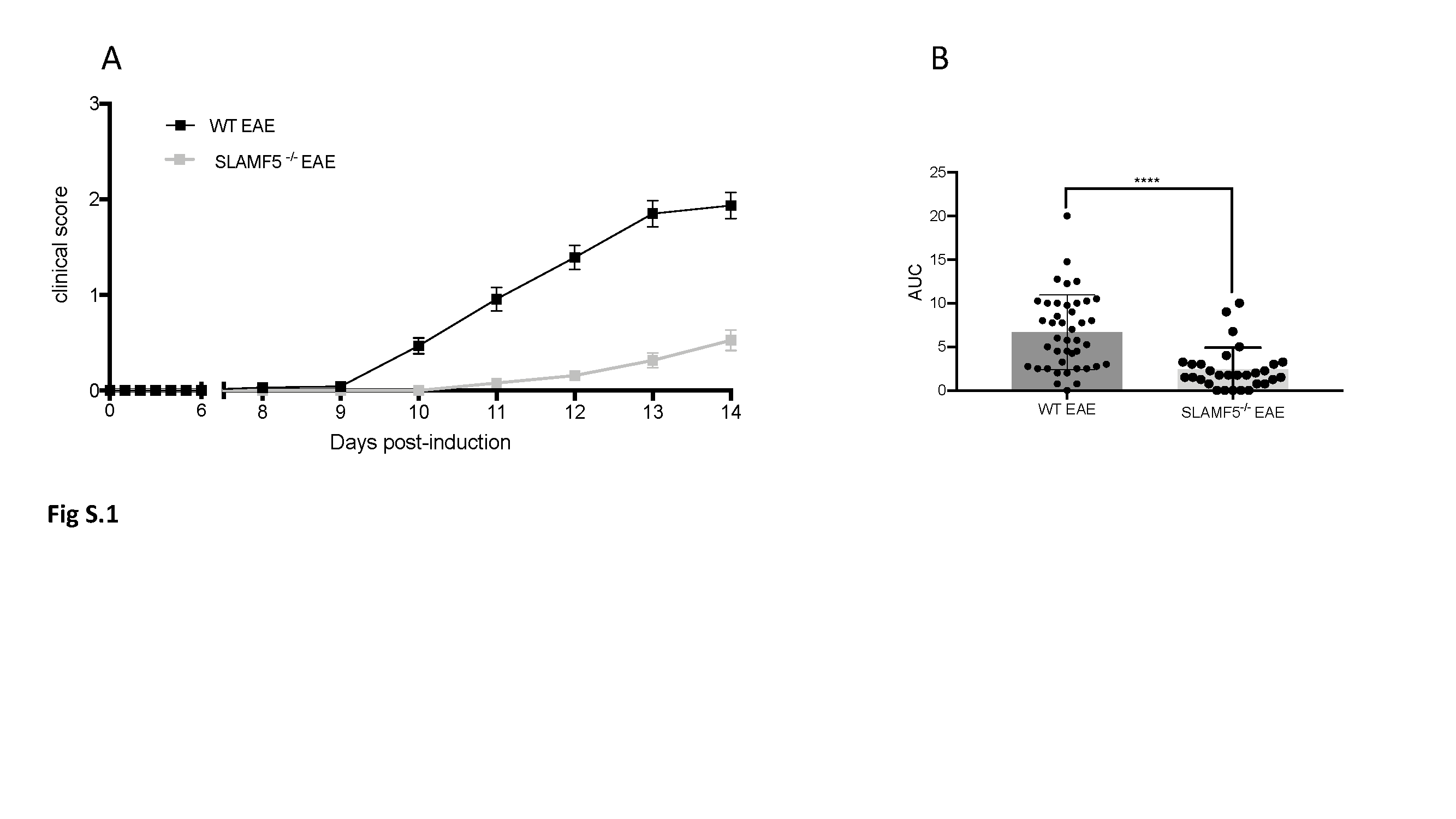

Supplement: S1 Fig — EAE (MOG35-55) was induced in WT and in SLAMF5-deficient mice. (A) Daily Mean clinical scoring of the disease. (B) Bar graph shows the Area Under the Curve of the clinical score. Mann-Whitney test (EAE induced WT group, n = 52; EAE-induced SLAMF5−/− group, n = 38). (*P < 0.05, **P < 0.01, ***P < 0.001, ****P < 0.0001). Data are shown as mean ± SD. The data underlying this figure can be found in S1 Data. (TIFF) [file pbio.3003373.s001.tiff]

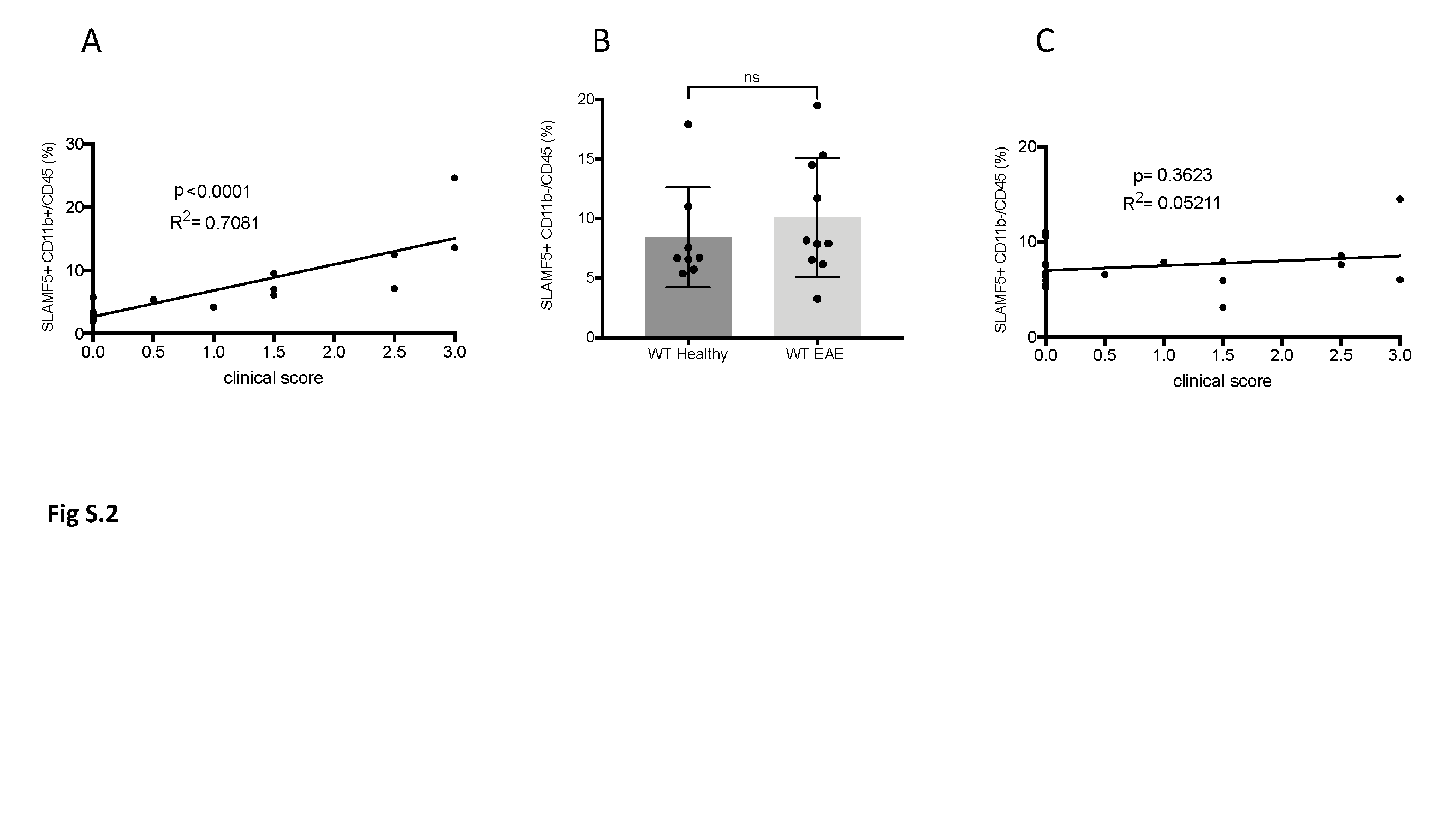

Supplement: S2 Fig — EAE (MOG35-55) was induced in WT and in SLAMF5-deficient mice. At day 15, the mice were sacrificed and their brains were excised. Their brains were processed, and immune cells were isolated, stained and analyzed by FACS. Dead cells were excluded from analysis by Zombie Live/Dead staining. Bar graphs showing (A) Correlation between SLAMF5 expression in CD45+ CD11b+ myeloid cells in the brain and disease severity(n = 18). (B) Percent expression of SLAMF5 in the CD45+ CD11b− non-myeloid cells in the brain (Healthy WT n = 8; EAE WT n = 10). (C) Correlation between the SLAMF5 expression in CD45+ CD11b− non-myeloid cells in the brain and disease severity (n = 18). Graphs show three independent determinations. Two-tailed unpaired Student t test with 95% confidence levels. (*P < 0.05, **P < 0.01, ***P < 0.001, ****P < 0.0001). Data are shown as mean ± SD. The data underlying this figure can be found in S1 Data. (TIFF) [file pbio.3003373.s002.tiff]

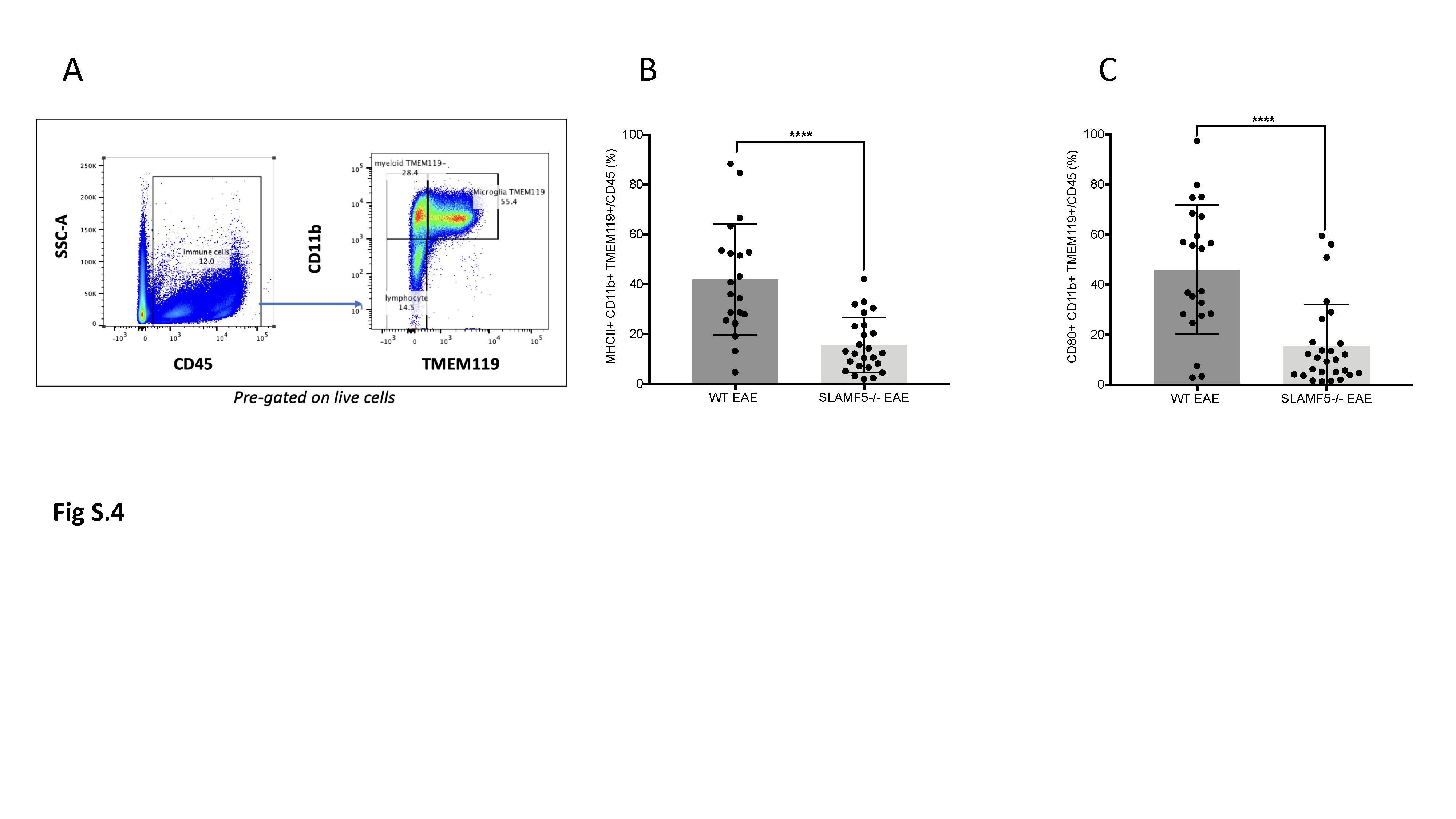

Supplement: S4 Fig — EAE (MOG35-55) was induced in mice. At day 15, the mice were sacrificed, and their brains were excised. The brains were processed and immune cells were isolated, stained and analysed by FACS. Dead cells were excluded from analysis by Zombie Live/Dead staining. (A) Representative plot showing gating strategy for brain suspension cells: CD45 was used to identify immune cells, CD11b for myeloid cells, and TMEM119+ for a specific population of microglial cells. Bar graphs show (B) the expression of MHCII in CD45+ CD11b+ TMEM119+ microglial cells (WT n = 20; SLAMF5−/− n = 25). (C) the expression of CD80 in CD45+ CD11b+ TMEM119+ microglial cells (WT n = 22; SLAMF5−/− n = 27). Two-tailed unpaired Student t test with 95% confidence levels. (*P < 0.05, **P < 0.01, ***P < 0.001, ****P < 0.0001). Data are shown as mean ± SD. The data underlying this figure can be found in S1 Data. (TIFF) [file pbio.3003373.s004.tiff]

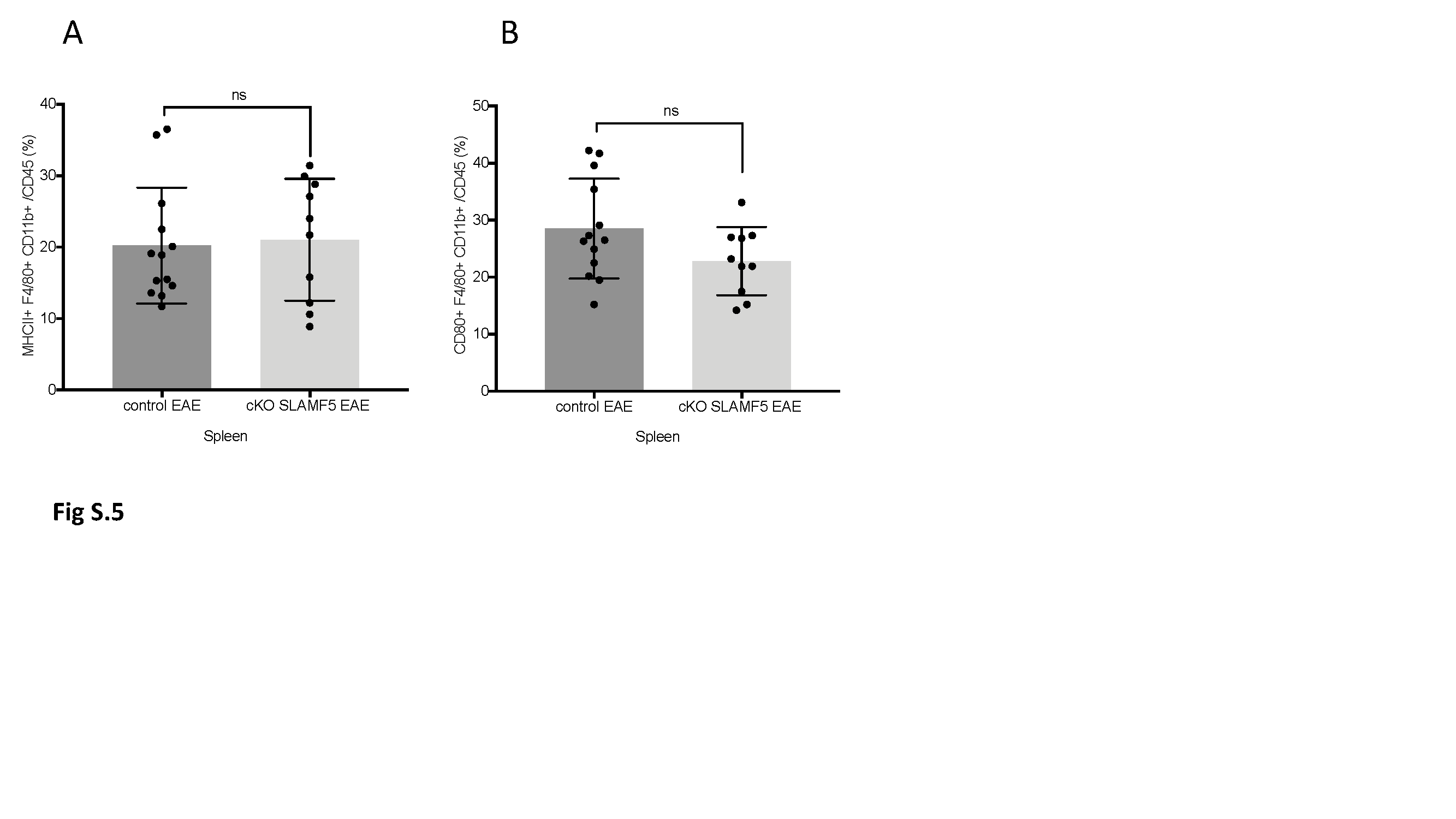

Supplement: S5 Fig — EAE (MOG35-55) was induced in control and CX3CR1 cre SLAMF5 flox mice. At day 15, the mice were sacrificed, and their spleens were excised. The spleens were processed, and immune cells were isolated, stained and analyzed by FACS. Dead cells were excluded from analysis by Zombie Live/Dead staining. Bar graphs show (A) the expression of MHCII in F4/80+ CD11b+ myeloid cells (control EAE n = 13; cKO SLAMF5 EAE −/− n = 10). (B) the expression of CD80 in F4/80+ CD11b+ myeloid cells (control EAE n = 13; cKO SLAMF5 EAE −/− n = 10). Two-tailed unpaired Student t test with 95% confidence levels. (*P < 0.05, **P < 0.01, ***P < 0.001, ****P < 0.0001). Data are shown as mean ± SD. The data underlying this figure can be found in S1 Data. (TIFF) [file pbio.3003373.s005.tiff]

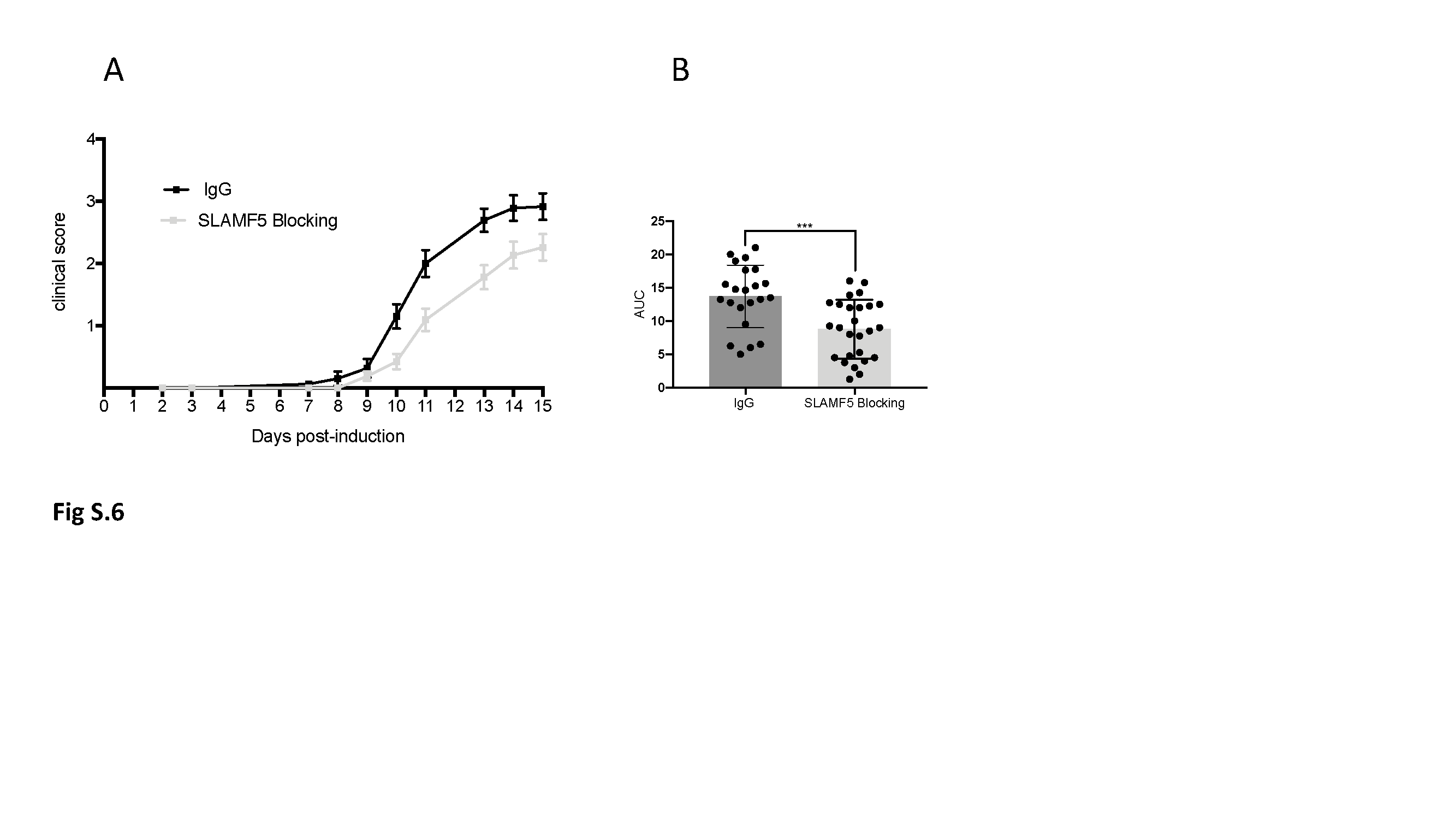

Supplement: S6 Fig — EAE (MOG35-55) was induced in WT mice and mice were treated with 3 injections, I.V, at days 7,9, and 12 postinduction with 150 μg of the SLAMF5 blocking antibody or the IgG control. Mice were monitored daily for 15 days. (A) Graph shows the Daily Mean clinical scoring of the disease. (B) Bar graph shows the Area Under the Curve of the clinical score. (SLAMF5-blocking n = 22; IgG n = 26). Mann–Whitney test. (*P < 0.05, **P < 0.01, ***P < 0.001, ****P < 0.0001). Data are shown as mean ± SD. The data underlying this figure can be found in S1 Data. (TIFF) [file pbio.3003373.s006.tiff]

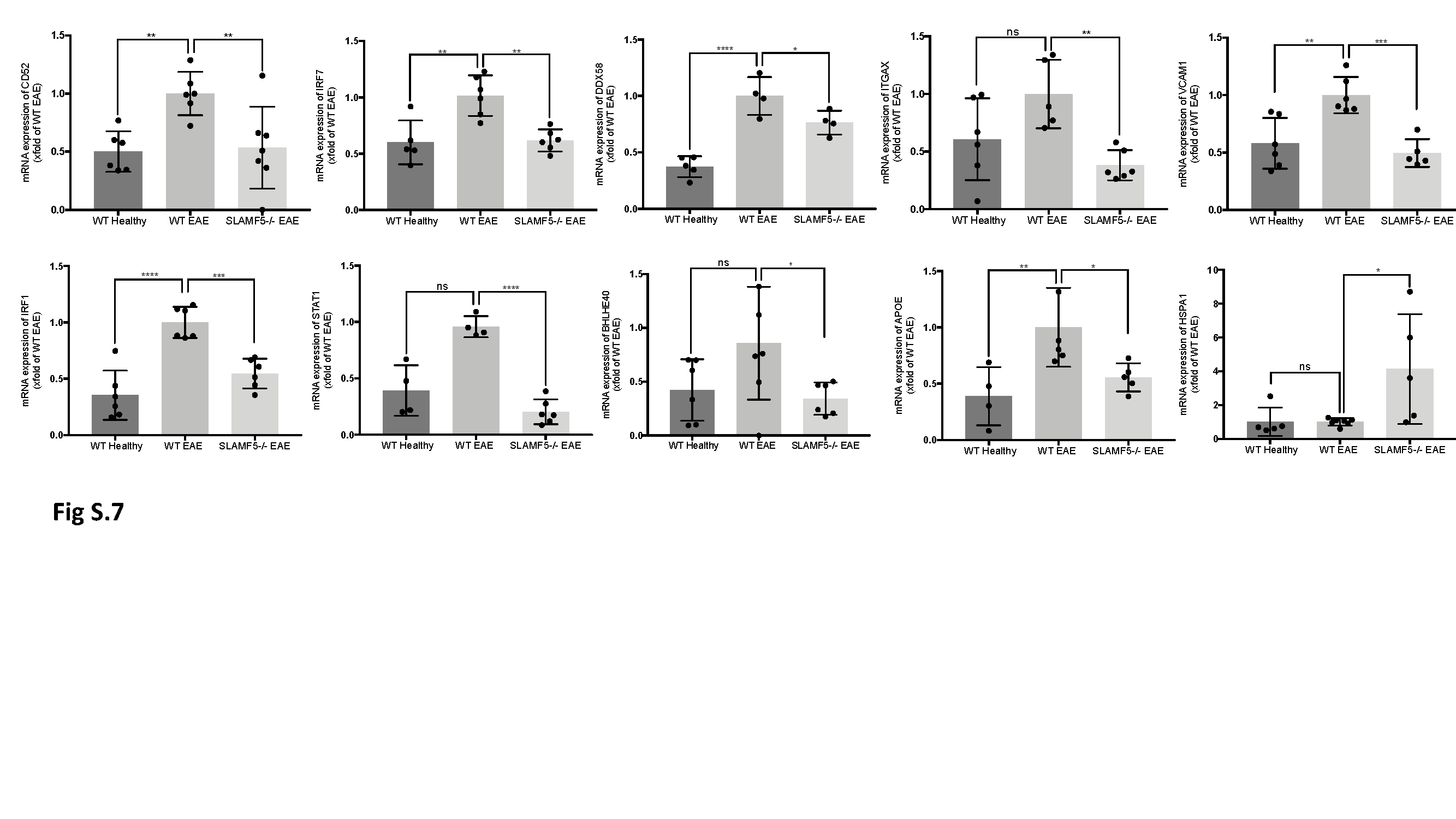

Supplement: S7 Fig — EAE (MOG35-55) was induced in WT and SLAMF5−/− mice. At the peak of the disease, the mice were sacrificed, and their brains were excised. The brains were processed, and immune cells were isolated, stained for dead cells, and labeled with anti-CD45 and anti-CD11b antibodies. The double positive population was sorted. RNA was isolated and cDNA was synthesized. The expression levels of genes were determined by qRT-PCR. Data are presented as fold change relative to the WT EAE group. Graphs show two independent determinations. Ordinary one-way ANOVA with Dunnett multiple comparison tests. (*P < 0.05, **P < 0.01, ***P < 0.001, ****P < 0.0001). Data are shown as mean ± SD. The data underlying this figure can be found in S1 Data. (TIFF) [file pbio.3003373.s007.tiff]

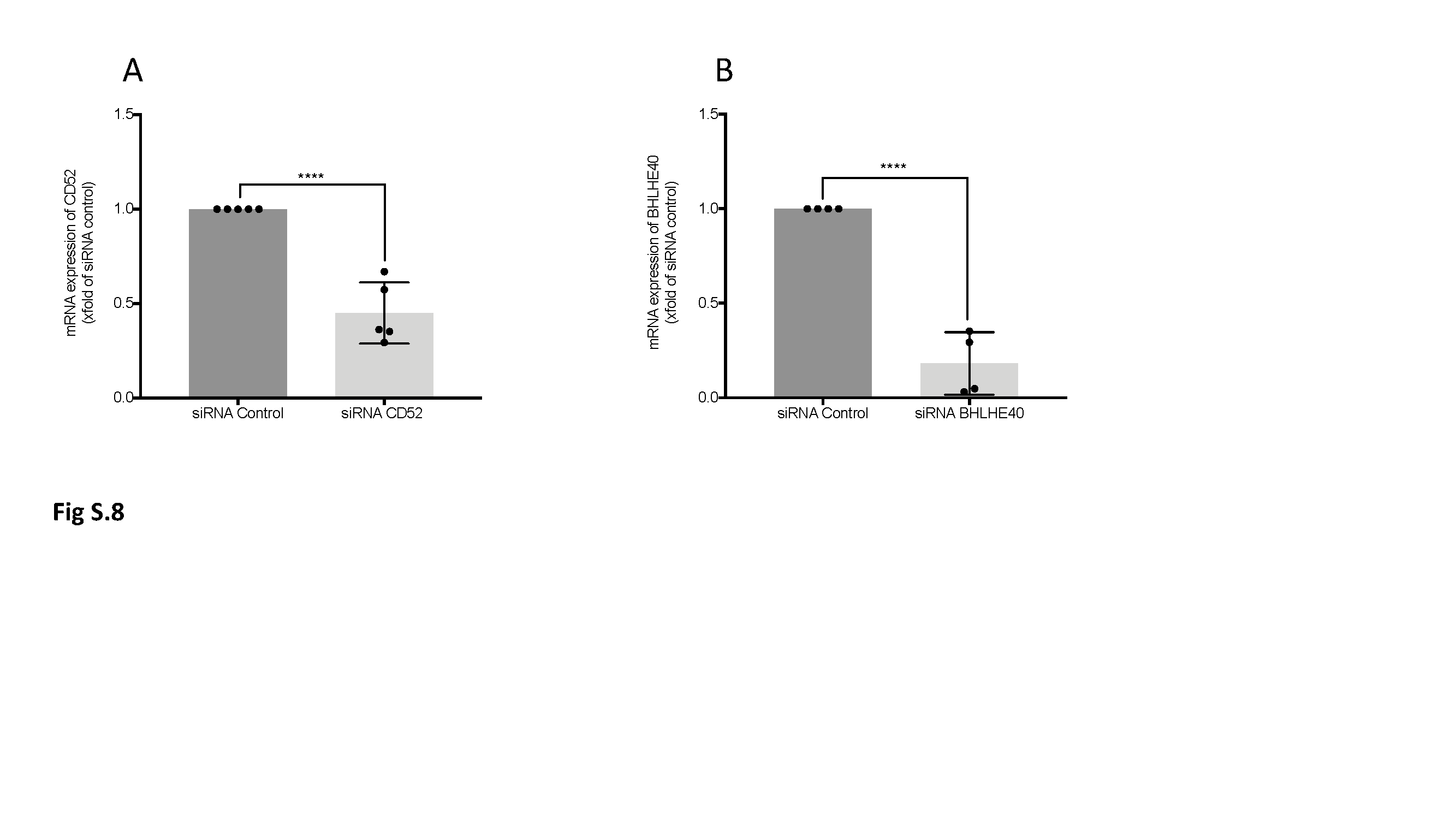

Supplement: S8 Fig — (A) N9, murine microglia cell line cells, were electroporated with CD52 siRNA or Control siRNA. RNA was isolated and cDNA was synthesized. The expression levels of CD52 were determined by qRT-PCR. Bar graph showing mRNA level of CD52 in control siRNA (n = 5), CD52 siRNA (n = 5). Graphs show two independent determinations. (B) N9, murine microglia cell line cells, were electroporated with BHLHE40 siRNA or Control siRNA. RNA was isolated and cDNA was synthesized. The expression levels of BHLHE40 were determined by qRTPCR. Bar graph showing mRNA level of BHLHE40 in control siRNA (n = 4), BHLHE40 siRNA (n = 4). Graphs show two independent determinations. Data are presented as fold change relative to the siRNA control group, which was normalized to a value of 1. Two-tailed unpaired Student t test with 95% confidence levels. (*P < 0.05, **P < 0.01, ***P < 0.001, ****P < 0.0001). Data are shown as mean ± SD. The data underlying this figure can be found in S1 Data. (TIFF) [file pbio.3003373.s008.tiff]

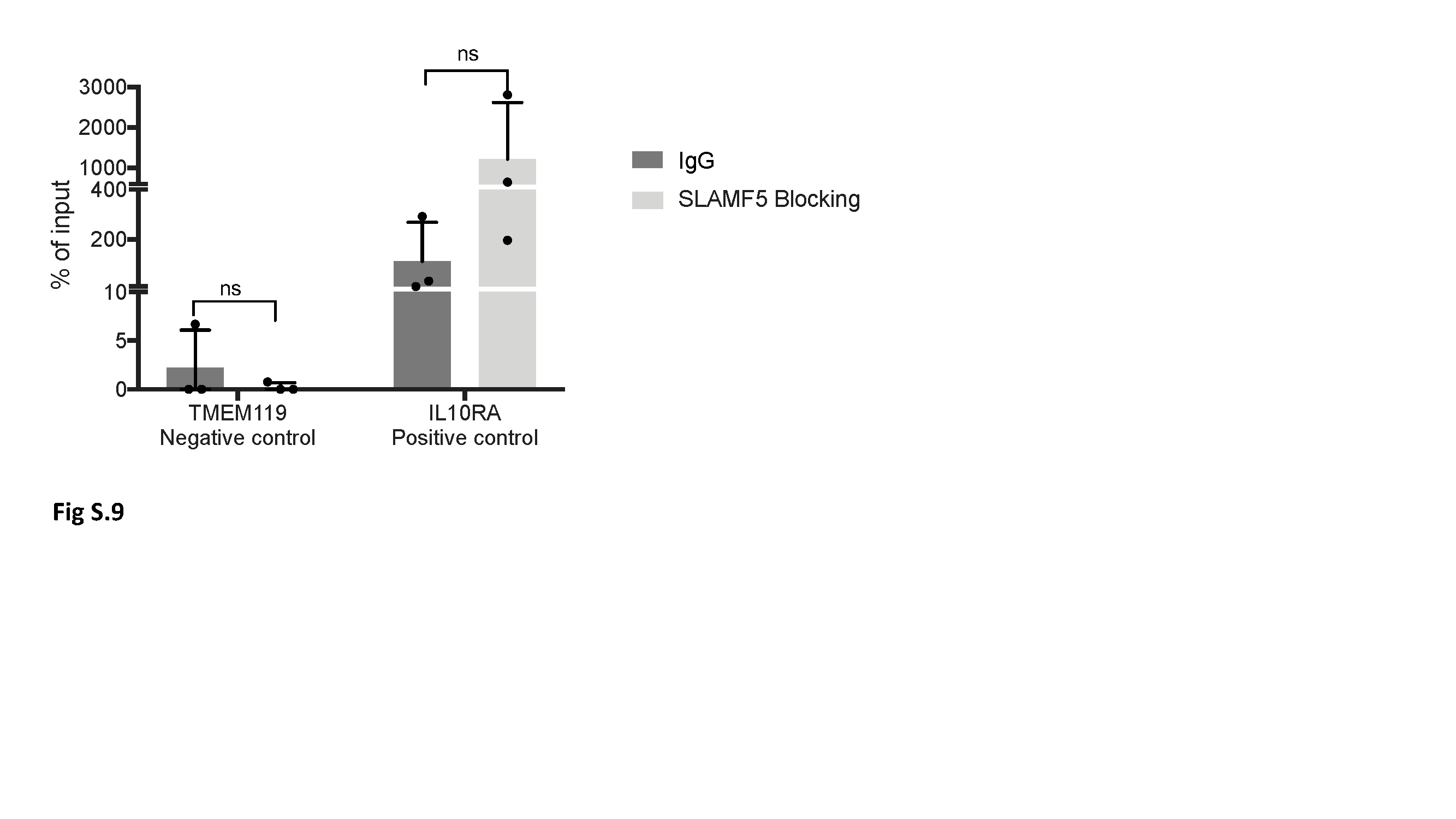

Supplement: S9 Fig — EAE (MOG35-55) was induced in WT mice. At the peak of the disease, the mice were sacrificed, and their brains were excised. The brains were processed, and immune cells were isolated, stained for dead cells, and labeled with anti-CD45 and anti-CD11b antibodies. The double positive population was sorted. The sorted cells were incubated with SLAMF5 blocking antibody or with the isotype control for 1 h. Then, cells were harvested, and ChIP was performed. Binding of BHLHE40 to the promoter area of the TMEM119 and IL10RA genes were determined by RT-qPCR. Graph presents the percent enrichment of the input. Two-tailed unpaired Student t test with 95% confidence levels. (*P < 0.05, **P < 0.01, ***P < 0.001, ****P < 0.0001). Data are shown as mean ± SD. The data underlying this figure can be found in S1 Data. (TIFF) [file pbio.3003373.s009.tiff]

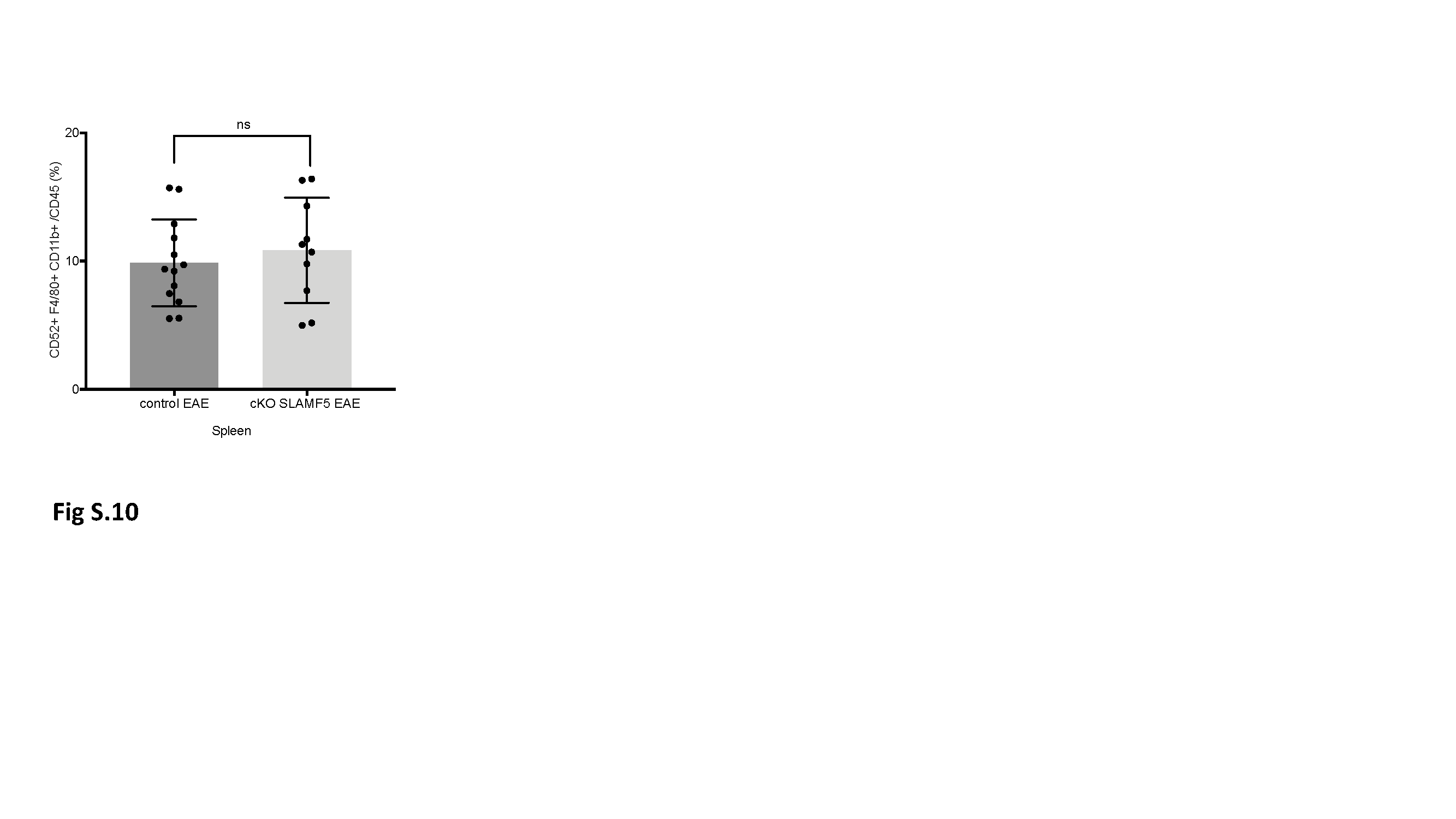

Supplement: S10 Fig — EAE (MOG35-55) was induced in control and CX3CR1 cre SLAMF5 flox mice. At day 15, the mice were sacrificed, and their spleens were excised. The spleens were processed, and immune cells were isolated, stained and analyzed by FACS. Dead cells were excluded from analysis by Zombie Live/Dead staining. Bar graph shows the expression of CD52 in F4/80+ CD11b+ myeloid cells (control EAE n = 13; cKO SLAMF5 EAE −/− n = 10). Two-tailed unpaired Student t test with 95% confidence levels. (*P < 0.05, **P < 0.01, ***P < 0.001, ****P < 0.0001). Data are shown as mean ± SD. The data underlying this figure can be found in S1 Data. (TIFF) [file pbio.3003373.s010.tiff]

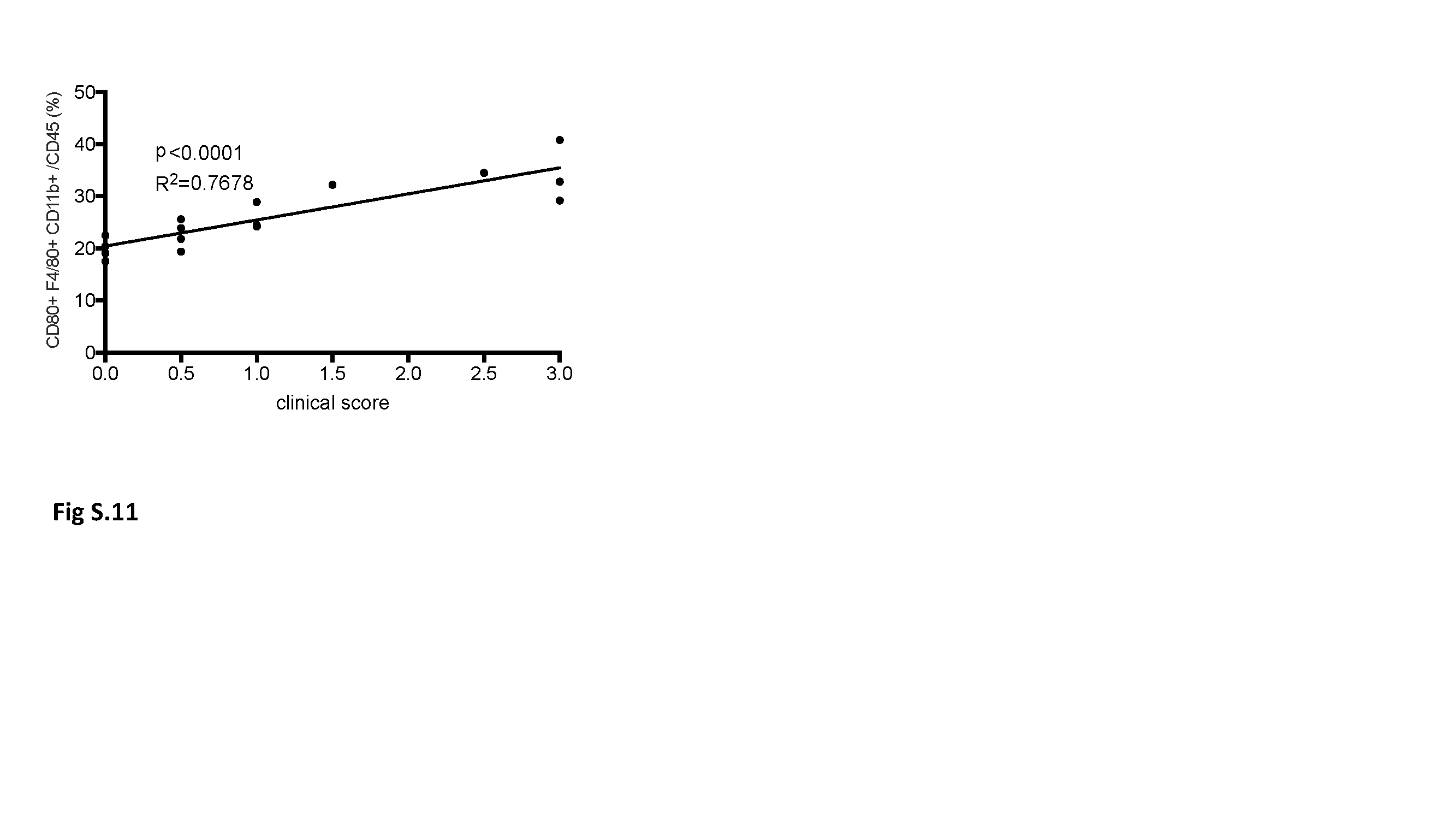

Supplement: S11 Fig — EAE (MOG35-55) was induced in WT and in SLAMF5-deficient mice. On day 15, the mice were sacrificed and their spleens were excised. Immune cells were isolated, stained and analyzed by FACS. Dead cells were excluded from analysis by Zombie Live/Dead staining. Representative bar graph shows the correlation between the CD80 expression on CD11b+ F4/80 myeloid cells in the spleen and the disease severity (n = 14). Graphs show four independent determinations. The data underlying this figure can be found in S1 Data. (TIFF) [file pbio.3003373.s011.tiff]

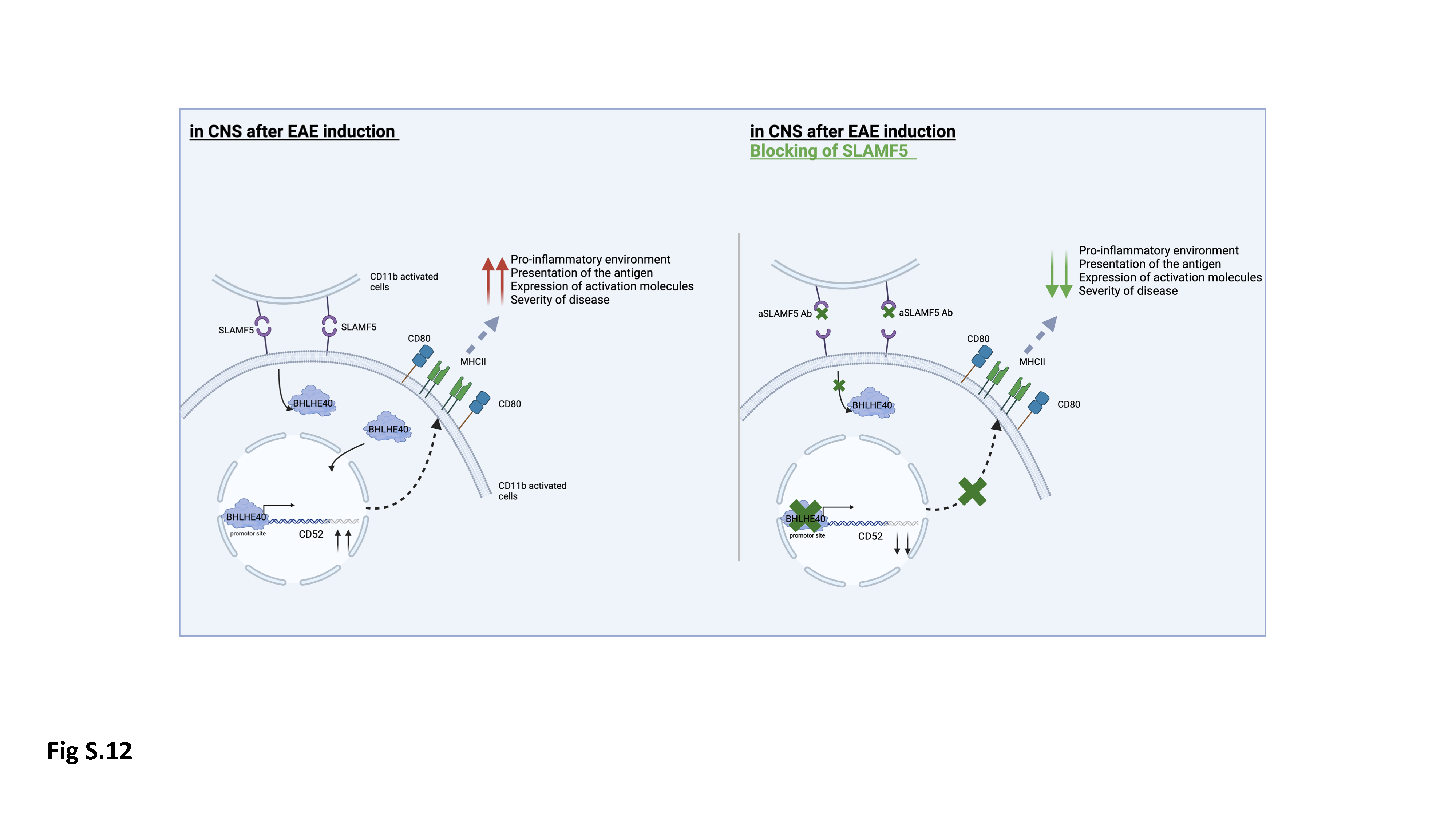

Supplement: S12 Fig — Using Biorender.com. (TIFF) [file pbio.3003373.s012.tiff]
