# Supplementary material for: The immune receptor SLAMF5 regulates myeloid-cell mediated neuroinflammation in multiple sclerosis
Source: PLoS Biol. 2025 Sep 8;23(9):e3003373. doi: 10.1371/journal.pbio.3003373 (PMC12431667; doi:10.1371/journal.pbio.3003373)
Supplement: Attachment — Submitted filename: response to the reviewer 26.5.25.docx [file pbio.3003373.s017.docx]

May 11^th^, 2025

Dear Melissa,

Thank you for your email from February 7th regarding our manuscript, “SLAMF5 Regulates Myeloid-Cell Mediated Neuroinflammation in Multiple Sclerosis.” We are gratified that the reviewers found substantial merit in our study. Nevertheless, the referees raised issues that they felt precluded publishing our manuscript in its present form. We have now addressed these issues in our revised manuscript.

More specifically,

**Reviewer 1**

1. **A key concern is that the authors already provided data that global SLAMF5-KO and pharmacologically treating mice with SLAMF5 blocking antibodies reduces EAE severity (Radomir et al Nature Communications 2021). The authors present very similar data in the current manuscript, Fig 1, Fig 3A showing a similar phenotype.**

The paper by Radomir et al. describes the role of SLAMF5 in regulating the function of peripheral lymphocyte populations, particularly Bregs. The current paper focuses on the role of this receptor in regulating the function of myeloid cells in the brain. Therefore, there is no overlap between these two studies. The only shared data between the studies pertains to the role of SLAMF5 in disease development, which serves as a basis for both studies. We could have simply described the results of the previous study, but we chose to present the data here to facilitate reader comprehension. However, due to the reviewer’s comments and to avoid any repetition with our previous study, we have moved these graphs to the supplementary section.

**The new and exciting data comes from Fig 2D showing that conditional SLAMF5-KO in Cx3cr1+ cells protect from EAE pathogenesis. However, the precise mechanisms in this strain are not followed up. There is no histopathology in this strain, RNA-seq analyses from sorted microglia, or additionally insights from this line of work. Therefore, as is, the in vivo studies are rather descriptive and should be expanded on.**

We appreciate the reviewer’s suggestion and agree that elucidating the mechanism of action of SLAMF5 is critical. However, we are unclear as to why the reviewer perceives a mechanistic difference between the full knockout (KO) and the conditional knockout (cKO) models. In both cases, SLAMF5 expression is effectively downregulated in sorted microglial cells, and the comparisons are made between cells that express or do not express SLAMF5.

While the cKO model introduces SLAMF5 deletion in a temporally controlled manner via tamoxifen administration, the recombination efficiency can vary and is time-dependent. To ensure maximal and uniform deletion for transcriptomic analysis, we performed RNA-seq on myeloid cells from full KO mice, where SLAMF5 is constitutively absent. This approach allowed us to confidently analyze the downstream gene expression changes with minimized variability.

To directly demonstrate that the downstream signaling cascade regulated by SLAMF5 is consistent across models, we analyzed the expression of SLAMF5-regulated genes identified in RNA-seq from full KO mice across several systems. As shown in the revised manuscript (new Fig. 4E), SLAMF5 regulates multiple genes in brain myeloid cells, including *CD52, Irf7, Irf1, Ddx58, Itgax, Vcam1, Stat1, Bhlhe40,* and *Hspa1b*—genes known to influence myeloid cell activation and EAE pathogenesis.

We next assessed the expression of these genes in additional models, including SLAMF5 cKO mice (Fig. 5A) and N9 microglial cells (Fig. 5B). In both cases, gene expression patterns mirrored those observed in the full KO model, confirming that SLAMF5 regulates a consistent transcriptional program across models. We focused particularly on *CD52*, due to its clinical relevance in MS. Reduced *CD52* expression was observed in cKO mice (Fig. 5 E–F), SLAMF5 antibody-treated mice (Fig. 5 G–H), primary microglia (Fig. 5 I–J), and N9 cells (Fig. 5 K–L).

Together, these results indicate that the SLAMF5-induced transcriptional cascade identified in RNA-seq is conserved across genetic and antibody-mediated models of SLAMF5 deficiency. Therefore, we respectfully disagree with the interpretation that our findings are merely descriptive. Rather, we believe our integrated analysis of KO, cKO, in vitro models, and pharmacologic inhibition comprehensively defines the downstream effects of SLAMF5 in CD11b⁺ myeloid cells.

However, to strengthen these results, as suggested by the reviewer, we have expanded our analysis of the cKO cells as follows:

1.	Histopathology: We performed spinal cord histology on EAE-induced cKO and control mice, which revealed significantly reduced lymphocyte infiltration in the cKO animals, supporting a protective effect in this model (new Fig. 2N).

2.	Naive State Comparison: We also examined the expression of activation markers in myeloid cells from naive (non-immunized) mice. No significant differences between cKO and control animals were observed, suggesting that SLAMF5 plays a role specifically during inflammation rather than in homeostatic conditions (new Fig. 2 K and M).

**2. The authors claim that therapeutic administration of SLAMF5 antibody did not impact MHCII, CD80 or other markers on myeloid cells due to its ability to be able to cross the blood-brain barrier. It is widely known that the EAE model has extensive BBB disruption through the course of disease and thus this is not a likely factor for the lack of its effect on these markers. A more unbiased approach, such as performing RNA-seq or CITE-seq on sorted cells from the Antibody or conditional KO mice would be more informative than queries on a select few markers.**

Our conclusion regarding limited antibody access to CNS-resident myeloid cells was based on the limited in vivo effect of the IV-injected antibody (Fig 3 A-B) versus dramatic effect its (ICV) injection experiments (Fig 3 L-R). In these studies, bypassing the BBB enabled the SLAMF5-blocking antibody to reach CNS-resident cells and resulted in reduced expression of MHCII and CD80—an effect not observed with systemic delivery. These findings informed our interpretation that the antibody’s functional reach is limited by its ability to access the CNS microenvironment, despite partial BBB compromise in EAE.

As the reviewer suggested, we used an unbiased approach to identify several pathways regulated by SLAMF5 in our RNA-seq results. As explained above, in answer no 1, this approach led to the identification of distinct pathways, as shown in the new. To directly demonstrate that the downstream signaling cascade regulated by SLAMF5 is consistent across models, we analyzed the expression of SLAMF5-regulated genes identified in RNA-seq from full KO mice across several systems. As shown in the revised manuscript (new Fig. 4E), SLAMF5 regulates multiple genes in brain myeloid cells, including *CD52, Irf7, Irf1, Ddx58, Itgax, Vcam1, Stat1, Bhlhe40,* and *Hspa1b*—genes known to influence myeloid cell activation and EAE pathogenesis.

We next assessed the expression of these genes in additional models, including SLAMF5 cKO mice (Fig. 5A) and N9 microglial cells (Fig. 5B). In both cases, gene expression patterns mirrored those observed in the full KO model, confirming that SLAMF5 regulates a consistent transcriptional program across models. We focused particularly on *CD52*, due to its clinical relevance in MS. Reduced *CD52* expression was observed in cKO mice (Fig. 5 E–F), SLAMF5 antibody-treated mice (Fig. 5 G–H), primary microglia (Fig. 5 I–J), and N9 cells (Fig. 5 K–L).

The results of these studies show that blocking SLAMF5 regulates the same pathways identified in the RNA-seq analysis. Therefore, we are uncertain about the added benefit of performing RNA-seq again on the anti-SLAMF5-treated cells.

**3. The RNA-seq data presented in Fig 5 is difficult to follow. The heat map shows 1 mouse at score 0 (healthy), four mice at score 0.5, 1 mouse at score 1 and two mice at score 3. How were stats performed with these numbers? A PCA plot showing the sample distribution along with all of the replicates per group are needed to understand the data differences. Fig 5D is comparing select genes across genotypes with varying scores and no healthy animals.**

We appreciate the reviewer’s careful evaluation of our RNA-seq data and agree that a PCA plot is important to clarify sample distribution. In the revised manuscript, we have added a PCA plot (now Fig. 4 A), which shows the clustering of individual samples based on gene expression profiles.

We would like to clarify how the statistical analysis was performed. Differentially expressed genes (DEGs) were defined by an adjusted p-value < 0.05, an absolute fold change > 1.5, and a minimum read count of 30 in at least one sample.

The PCA revealed distinct clustering patterns corresponding to disease severity. Wild-type (WT) mice with higher clinical scores (score 3) clustered together and separately from WT mice with milder disease (scores 0.5–1). Importantly, SLAMF5 KO mice, which displayed relatively uniform disease severity, clustered together as well, suggesting consistent transcriptional changes in this group.

Despite some variability in disease scores among WT mice, we identified a robust set of 395 genes significantly downregulated and 58 genes upregulated in SLAMF5 KO myeloid cells compared to WT, underscoring the broad regulatory impact of SLAMF5 during neuroinflammation. Although no fully healthy animals were included in the DEG analysis, the internal consistency of the KO group and stratification of WT animals by disease severity in the PCA support the validity of the conclusions drawn. We include this explanation in the Results section.

**4. It is unclear how many independent experiments, number of differentiations from iPSC to iMicroglia, and how many iPSC lines were used throughout the study.**

Throughout the study, we used a well-characterized iPSC line from the Knockout Laboratory (KOLF2.1J, wild type) [1-3]. A total of three independent experiments were performed: the first experiment was conducted using initial batch of iPSCs, and the subsequent two experiments were performed using a second batch of cells. These details have now been added to the Methods section of the revised manuscript for clarity.

**5. Does blocking SLAMF5 on microglia or macrophages affect their antigen presentation capacity to T cells?**

It was previously shown that SLAMF5-deficient mice showed no phenotype except for the regulation of germinal center formation [4]. We discussed the antigen presentation pathway in the revised manuscript. In addition, our findings support the idea that blocking SLAMF5 in myeloid cells affects their ability to support T cell activation. In both knockout and conditional SLAMF5 knockout models, we observed a marked reduction in the accumulation of CNS lymphocytes and decreased IFN-γ production by CD4⁺ T cells. These results suggest that SLAMF5 promotes a pro-inflammatory environment, likely during neuroinflammation (Fig. 1 G-J and Fig. 2 N-Q).

**Reviewer 2**

Major points:

**In the introduction, the importance of lymphocyte and myeloid cell phenotypes for the severity and evolution of the disease could be mentioned. This aspect should also be discussed when discussing the data obtained in this study.**

We added in the introduction the missing information.

**- p.4, last paragraph: antigen presentation, amplification of inflammation and modulation of adaptive immune cues are not restricted to recruited macrophages and monocytes, but are features shared with microglia. This should be mentioned.**

We added the missing information.

**- p.5: "SLAMF5 …is broadly expressed on most commune immune cell subsets". This point could be detailed, as the present sentence is very vague and this question is of importance for the subject explored by the authors.**

We elaborated on this topic in the introduction.

**-There is a global lack of relevant information (description of samples, timings, etc) in the material and methods. For example, the effect, half-life … of the blocking antibody could be described more thoroughly to facilitate the understanding of the protocol of injection used and the results obtained. Regarding RNA sequencing, could the authors detail in the material and methods the exact nature of the samples used?**

We added the missing information throughout the text.

**Also, the authors used LPS to activate microglial cells though stimulation by well-chosen cytokines could have been more relevant for MS. This could be discussed.**
We chose LPS to activate N9 microglial cells based on prior studies, including those by *Cunha et al. (2016)*[5], which demonstrate that LPS stimulation can effectively mimic M1 polarization—a phenotype commonly observed in neuroinflammatory diseases like EAE. Specifically, LPS-induced activation leads to upregulation of MHC-II and iNOS, as well as downregulation of markers such as arginase 1, FIZZ1, and CX3CR1, which are indicative of M1 polarization. We added this information to the manuscript.

In our own experiments, we observed that LPS treatment of N9 microglial cells resulted in an upregulation of MHC-II and CD80 expression, mirroring the changes observed in EAE mice. These results further support the use of LPS as a relevant stimulus for microglial activation in our study. However, we acknowledge the reviewer’s point and will discuss this choice more explicitly in the revised manuscript, clarifying the rationale for using LPS as an activation signal and noting the potential relevance of cytokine-driven activation pathways for future studies in MS.

We discussed this point in the results section.

**- p. 10: As the symptom appearance is delayed in the SLAMF5-/-, it would be more relevant to compare the EAE scores 5 days after symptom onset (day 15 for controls vs day 18 for SLAMF5-/-) than comparing them all at day 15, or comparing them at the peak of the disease for each condition. Figure 1C could be presented prior to Figure 1.**

Our EAE experiments are constrained by institutional animal care regulations (IACUC), which require euthanasia when any mouse reaches a clinical score of 3, due to the associated weight loss exceeding 20%. Therefore, we are not able to prolong the experiments much longer since the score of the WT mice is high.

In addition, to ensure consistency in sample processing, particularly in preparing single-cell suspensions from tissues, we decided to sacrifice all mice at the same time point. This was critical to minimize technical variability in cell isolation, which can significantly impact downstream analyses such as flow cytometry and gene expression. In addition, performing flow cytometry staining for all samples simultaneously allowed us to minimize variability introduced during antibody staining and acquisition, ensuring more reliable comparisons across genotypes and conditions. Variability in timing could introduce differences in cell viability or activation states unrelated to genotype or disease severity.

We clarified this rationale in the revised manuscript and reordered the figures.

**- p. 11. To my knowledge, some activated microglia still express TMEM119. I would thus write "TMEM119 positive microglia" rather than "TMEM119 positive homeostatic microglia". Also, the equivalence between Cd11b- and lymphocytes is unclear to me, this should be (re)explained here.**

We have corrected the text to refer to “TMEM119⁺ microglia,” as it is indeed recognized that TMEM119 can be retained on some activated microglia.

Regarding T cell identification, we clarify that our initial gating strategy was based on CD45 expression to select for all leukocytes, followed by CD11b to separate myeloid cells (CD11b⁺) from lymphocytes (CD11b⁻). Within the CD45⁺CD11b⁻ population, we then used CD3, CD4, and CD8 markers to identify total T cells and distinguish between CD4⁺ and CD8⁺ T cell subsets. This revised explanation has been added to text.

**The lower number of T cells could be due to lesser infiltration, or retention or survival. This should be shown or at least discussed. The conclusion regarding Th1 as being the prominent T cell type associated to the milder form of the disease in SLAMF5 deficient mice should be strengthened or presented more cautiously. Is the reduction in Th1 due to cell autonomous or non-cell autonomous mechanisms in SLAMF5 mutant? Is this known?**

We have revised the description of the Th1 cells to be more cautious. Our results show that CD4⁺ T cells in SLAMF5-deficient mice produce less IFN-γ, suggesting decreased polarization of the cells to activated T cells. However, whether this reduction is due to a cell-autonomous effect in T cells or is driven by changes in the myeloid environment (e.g., altered antigen presentation or cytokine production) remains to be determined. It was previously shown that SLAMF5 deficiency has no effect on T cell survival, activation and function [4]. In addition, we added to the current manuscript Fig 2N showing that reduced expression of SLAMF5 in microglia cells, lowered the accumulation of T cells in the brain. Therefore, it can be hypothesized that the environment lacking SLAMF5 regulates cell accumulation and function. We discussed this issue in the discussion section.

**- p12: again, it would be better to compare the mean scores of animals with similar disease duration, or at the peak of the disease of each group. If the blocking antibody does not cross the blood-brain barrier, how does the antibody affect the severity of the disease? This should be discussed.**

As mentioned above, to avoid technical variation in downstream analyses, particularly in the preparation of single-cell suspensions from the brain and staining for flow cytometry, we sacrificed all animals in the experiment at the same time point once any mouse reached score 3. We have clarified this rationale in the revised manuscript.

Regarding the effect of the antibody, despite limited central CNS engagement, our previous study [6] showed that SLAMF5 plays an important role in the differentiation and function of IL-10-producing regulatory B cells in the periphery. The improvement in disease severity following systemic SLAMF5 blockade is therefore likely mediated through effects on peripheral immune regulation, including modulation of Breg and T cell responses. This would explain the impact on clinical scores even though no changes were observed in CNS-resident myeloid cell markers like MHCII and CD80. We have now discussed this point more clearly in the revised manuscript.

**The effect of the blocking antibody addition on cultured microglia activated with LPS is relatively limited regarding MCHII and CD80 activation. Could the authors comment on that?**

As for the in vitro experiments with LPS-stimulated N9 microglial cells, we observed strong upregulation of MHCII (~1.75-fold) and CD80 (~8.5-fold) upon LPS treatment. These elevations mimic the in vivo activation of microglia and antigen-presenting cells during EAE induction. Importantly, when we treated these LPS-activated microglial cells with the SLAMF5 blocking antibody, we found that both MHCII and CD80 expression were significantly reduced. The levels of MHCII in antibody-treated cells were comparable to those in unstimulated controls, and CD80 expression was markedly lower compared to LPS-treated cells (Fig. 3 C-F). This demonstrates that SLAMF5 plays an important role in modulating the activation of microglia and that blocking SLAMF5 has a significant effect and can dampen this activation. We have now included these findings, with the corresponding fold-change data, in the Results section for clarity.

**- p13: how can the ICV with SLAMF5 blocking antibody and the cKO of SLAMF5 have so drastic effects compared to the SLAMF5 mutant regarding EAE scores? This is a major concern regarding the models used in this study.**

The full KO mice have a systemic, lifelong loss of SLAMF5 expression, leading to a complete absence of SLAMF5 in all immune cells from early stage. This may cause compensatory mechanisms or other immune adaptations that could partially mitigate the disease severity over time.

In contrast, in the cKO model and blocking SLAMF5 in vivo, the loss or reduced function of SLAMF5 is achieved during the course of the disease, and therefore the compensatory mechanisms are not triggered. As a result, the effect on the disease in these models on disease progression are more pronounced.

**- How would the targeting of SLAMF5 in MS patients be an improvement compared to the existing approaches targeting CD52? What therapeutical approach could be used to target SLAMF5 in MS patients? Could there be increased risks for the patients by targeting SLAMF5 in case it is a more global regulator of immunity rather than targeting its downstream target CD52 specifically? In the discussion, the effect of CD52 specific blocking on EAE should be discussed.**
We thank the reviewer for these thoughtful points. In the revised manuscript we addressed the potential advantages and risks of targeting SLAMF5 in MS patients in the discussion section, with a comparison to existing approaches targeting CD52.

SLAMF5, as we show in our work, plays a role in modulating immune responses during disease progression, especially in myeloid cells such as microglia and macrophages. In the peripheral blood of MS patients, we showed that SLAMF5 blocking also induced lower level of HLA-DR, CD80 and CD52. Targeting SLAMF5 could provide a more targeted and potentially safer approach compared to broader immunosuppressive treatments like CD52 targeting. CD52-targeting therapies, such as alemtuzumab, broadly deplete both T and B lymphocytes, which can lead to wide-ranging immunosuppression and associated risks, including infections and secondary autoimmune conditions. In contrast, SLAMF5-targeted therapies could more specifically modulate the immune response within the brain and spinal cord, potentially reducing neuroinflammation without broadly affecting peripheral immunity.

Given SLAMF5's role in regulating myeloid cell activation, one potential approach could involve the use of SLAMF5 blocking antibodies that can specifically target SLAMF5 signaling in the CNS. This could be particularly beneficial in MS, where neuroinflammation mediated by activated microglia and macrophages plays a critical role in disease pathogenesis.

**Reviewer 3**

**1. There is some overlap in the results presented here and what was published in their Nature Communications paper (reference 34). In both studies they examined EAE in SLAMF5 null mutants, as well as in mice treated with a blocking SLAMF5 antibody. I would suggest removing the duplicative data, or at least moving this to the supplemental figures.**

The paper by Radomir et al. describes the role of SLAMF5 in regulating the function of peripheral lymphocyte populations, particularly Bregs. The current paper focuses on the role of this receptor in regulating the function of myeloid cells in the brain. Therefore, there is no overlap between these two studies. The only shared data between the studies pertains to the role of SLAMF5 in disease development that serves as a basis for both studies. We could have simply described the results of the previous study, but we chose to present the data here to facilitate reader comprehension. However, due to the reviewer’s comments and to avoid any repetition with our previous study, we have moved these graphs to the supplementary section.

**2. The data presented in Figure 1 examines various parameters of myeloid cell activation in the EAE-induced SLAMF5 null mutants. Much of this is repeated in Figure 2 with the myeloid cell specific SLAMF5 mutant EAE. I Would suggest removing the SLAMF5 null mutant results completely (i.e., all of Figure 1) and enhancing Figure 2 to include all assays examined with the null mutants with the myeloid cell specific mutants.**

We thank the reviewer for the suggestion, but we will that in order to be able to compare the results of the cKO mice and the blocking antibody, we need to keep Figure 1 with the full SLAMF5 null mutant data, as it provides essential context for the broader understanding of the study and the basis of our study. However, in Figure 2, we have regrouped the experiments for the myeloid cell-specific SLAMF5 mutant (cKO), incorporating additional assays, including naive state, histopathology, and T cell analysis. This arrangement allows us to highlight the distinct features of both the null and myeloid-specific mutants while ensuring the clarity and depth of the presented data.

**3. The study relies heavily on FACS analyses, including the analysis of expression levels of various myeloid cell activation markers. Their gene expression studies would be more convincing if an additional, independent approach (qPCR of the sorted cells) was included.**

To address the reviewer’s concern, we have sorted myeloid cells from EAE cKO and control mice and examined the gene expression in RT-qPCR of the markers that were regulated in the RNA-seq data from the full KO (Fig. 5A). Additionally, we performed RT-qPCR on LPS-treated N9 cells with the SLAMF5 blocking antibody or IgG control and analyzed the expression of myeloid cell activation markers (Fig. 5B). These complementary approaches strengthen our findings and provide a more comprehensive understanding of the gene expression dynamics in our study.

**4. The full EAE disease course (28 day) should be included for the myeloid cell-specific SLAMF5 mutants (Figure 2), as well as the animals injected intracerebrally with the SLAMF5 blocking antibodies (Figure 4).**

Unfortunately, in accordance with animal welfare protocols and the IACUC guidelines, we are unable to follow the full 28-day disease course for the myeloid cell-specific SLAMF5 mutants or the SLAMF5 blocking antibody-injected animals. Mice must be sacrificed when their disease score reaches 3, or if they experience weight loss greater than 20%. To minimize background in tissue preparation and FACS staining, we have made the decision to end the experiment when mice reach this score 3. However, we added Fig. 2H the monitoring of the cKO mice since the disease in these mice did not reach score 3. Our results show no significant change in the disease score in the extended monitoring.

**5. The authors statement that "SLAMF5 controls BHLHE40 binding to the CD52 promoter" appears overstated. SLAMF5 increases the expression of BHLHE40, such that there is more DNA binding to the CD52 transcriptional control region (Figure 7d), but the authors do not provide evidence for SLAMF5 regulation of DNA binding.**

We sorted CD11b+ cells from WT EAE mice and treated them with a blocking antibody against SLAMF5 or an isotype control antibody. (Fig. 6K) The results showed that blocking SLAMF5 reduced the amount of BHLHE40 binding to the CD52 promoter region, suggesting that the effect of SLAMF5 on BHLHE40 binding is likely due to its role in regulating BHLHE40 expression as seen in the RNA sequencing (Fig. 4E).

We have updated the manuscript to better reflect this nuance and ensure that our conclusions are clearly stated.

**6. For the DNA binding studies, it would be more convincing if negative controls and positive (known DNA binding partners of BHLHE40) controls were included. Does the CD52 transcriptional control region contain an E-box DNA sequence, which is a known binding target of BHLHE40?**

It is not known whether BHLHE40 binds to the control region of CD52. Jarjour et al. demonstrated that BHLHE40 regulates CD52 expression in large peritoneal macrophages, but they did not show direct binding of the transcription factor [7]. To address the reviewer’s concern, we have now included both negative and positive controls in our DNA binding assays. Specifically, TMEM119 was used as a negative control, and IL10RA, a known binding target of BHLHE40, was employed as a positive control (supplementary Fig 8). Ebox is a non-TF specific TF binding site. Therefore, even if we show that we have an EBOX sequence in the regulatory areas, it wouldn't necessarily say that BHLH40E is binding to it.

To be more specific, we utilized the Genomatix software, and used its "MatInspector" tool.

We've inserted the FASTA sequence of -500 bp/ CD52 Promotor / +500bp and analyzed it.

It recognized the gene that is regulated to be as CD52, then in the output we could detect the TF matrix family of BHLH40e the V$HSEF. More specifically, when we analyzed the data to find specific TFs and not just families, we were able to detect V$DEC1.01/V$DEC1.02 that are the BHLH40E TF.

We believe that the additional results and clarifications described here strengthen our study and further substantiate our conclusions. We would therefore appreciate your continued consideration of this manuscript for publication in Plos Biology.

Yours Sincerely,

Professor Idit Shachar

**References**

1. Haenseler W, Sansom SN, Buchrieser J, Newey SE, Moore CS, Nicholls FJ, et al. A Highly Efficient Human Pluripotent Stem Cell Microglia Model Displays a Neuronal-Co-culture-Specific Expression Profile and Inflammatory Response. Stem Cell Reports. 2017;8(6):1727-42. doi: 10.1016/j.stemcr.2017.05.017. PubMed PMID: 28591653; PubMed Central PMCID: PMCPMC5470330.

2. Eitan C, Siany A, Barkan E, Olender T, van Eijk KR, Moisse M, et al. Whole-genome sequencing reveals that variants in the Interleukin 18 Receptor Accessory Protein 3'UTR protect against ALS. Nat Neurosci. 2022;25(4):433-45. Epub 2022/04/02. doi: 10.1038/s41593-022-01040-6. PubMed PMID: 35361972; PubMed Central PMCID: PMCPMC7614916.

3. Pantazis CB, Yang A, Lara E, McDonough JA, Blauwendraat C, Peng L, et al. A reference human induced pluripotent stem cell line for large-scale collaborative studies. Cell Stem Cell. 2022;29(12):1685-702 e22. doi: 10.1016/j.stem.2022.11.004. PubMed PMID: 36459969; PubMed Central PMCID: PMCPMC9782786.

4. Cannons JL, Qi H, Lu KT, Dutta M, Gomez-Rodriguez J, Cheng J, et al. Optimal Germinal Center Responses Require a Multistage T Cell:B Cell Adhesion Process Involving Integrins, SLAM-Associated Protein, and CD84. Immunity. 2010;32(2):253-65. PubMed PMID: ISI:000275346000014.

5. Cunha C, Gomes C, Vaz AR, Brites D. Exploring New Inflammatory Biomarkers and Pathways during LPS-Induced M1 Polarization. Mediators Inflamm. 2016;2016:6986175. Epub 20161221. doi: 10.1155/2016/6986175. PubMed PMID: 28096568; PubMed Central PMCID: PMCPMC5209629.

6. Radomir L, Kramer MP, Perpinial M, Schottlender N, Rabani S, David K, et al. The survival and function of IL-10-producing regulatory B cells are negatively controlled by SLAMF5. Nat Commun. 2021;12(1):1893. Epub 2021/03/27. doi: 10.1038/s41467-021-22230-z. PubMed PMID: 33767202; PubMed Central PMCID: PMCPMC7994628.

7. Jarjour NN, Schwarzkopf EA, Bradstreet TR, Shchukina I, Lin CC, Huang SC, et al. Bhlhe40 mediates tissue-specific control of macrophage proliferation in homeostasis and type 2 immunity. Nat Immunol. 2019;20(6):687-700. Epub 20190506. doi: 10.1038/s41590-019-0382-5. PubMed PMID: 31061528; PubMed Central PMCID: PMCPMC6531324.
