# Supplementary material for: The immune receptor SLAMF5 regulates myeloid-cell mediated neuroinflammation in multiple sclerosis
Source: PLoS Biol. 2025 Sep 8;23(9):e3003373. doi: 10.1371/journal.pbio.3003373 (PMC12431667; doi:10.1371/journal.pbio.3003373)
Supplement: Attachment — Submitted filename: Answers to the reviewers 23.7.docx [file pbio.3003373.s019.docx]

July 17^th^, 2025

Dear Melissa Vázquez Hernández,

Thank you for your letter dated July 13th regarding our manuscript, *“SLAMF5 Regulates Myeloid Cell-Mediated Neuroinflammation in Multiple Sclerosis”*, authored by Laura Bellassen, Keren David, Bar Lampert, Avital Sarusi-Portuguez, Michael Tsoory, Jazz Lubliner, Michael Osherov, Ori Brener, Ron Milo, Eran Hornstein, Shirly Becker-Herman, and myself.

We are pleased that two of the reviewers recognize the good job done addressing their previous concerns, but Reviewer #1 still would like some aspects to be addressed. We have carefully considered the reviewer’s comments and revised the manuscript accordingly to address the issues of concern.

**Reviewer 1**

Reviewer comment 1:

**While the authors may consider there is "no overlap between these two studies"; two reviewers, myself and Reviewer 3, independently noted the data similarity between the authors prior study in Nat Commun.**

The paper by Radomir et al. describes the role of SLAMF5 in regulating peripheral lymphocyte populations, particularly regulatory B cells (Bregs). In contrast, the current study focuses on the role of SLAMF5 in modulating the function of myeloid cells within the brain. Therefore, there is no overlap between the two studies.

The only shared component is the use of SLAMF5 full knockout mice and mice treated intravenously with a SLAMF5-blocking antibody to assess disease development—an experimental foundation common to both studies. Importantly, the graphs presented here are not identical to those previously published; additional experiments have been conducted since the original publication, and the updated results are now included in the current study.

While we could have referred readers to the earlier publication, we chose to include the data here to enhance clarity and comprehension. These graphs have been placed in the Supplementary Figures (Fig. S1A and Fig. S6A).

**"we are unclear as to why the reviewer perceives a mechanistic difference between the full knockout (KO) and the conditional knockout (cKO) models. In both cases, SLAMF5 expression is effectively downregulated in sorted microglial cells, and the comparisons are made between cells that express or do not express SLAMF5."**

**The authors title and text claim a specific role for SLAMF5 in regulating myeloid-cell mediated neuroinflammation. To support this conclusion, assays using cell-type specific ablation are critical to inform on cell autonomous functions. The authors data in the global SLAMF5 KO (original Fig 1A-B) showed that animals begin to show EAE clinical symptoms by day 14. The cKO mice are largely (80% of the mice in Fig. 2H,I) protected from EAE disease based on the curve and AUC of zero. Based on the scores, the data suggest that myeloid-specific ablation has a unique difference than global KOs in EAE pathogenesis. In KO mice, given the authors performed flow and RNA-seq experiments on all CD45+CD11b+ cells, which in EAE includes many cell-types and activation states of each of them, cell-specific analysis is required to support such conclusions. How Slamf5 regulates myeloid cell mediated neuroinflammation in EAE remains unclear. In microglia or macrophages, is there any signaling pathways deregulated in Slamf5 dependent manner? The RNA-seq data suggest some, but functional validation studies are not explored, and thus the study remains descriptive as presented.**

We agree with the reviewer that dissecting the role of SLAMF5 specifically in myeloid cells is essential for understanding its contribution to EAE development. To address this, we generated conditional knockout (cKO) mice with SLAMF5 ablated in brain myeloid cells and monitored their behavior and cellular function. Our findings indicate that SLAMF5 expression in brain myeloid cells plays a critical role in both the initiation and progression of disease.

To define the functional impact of SLAMF5 in these cells, we sorted brain myeloid populations from full knockout (KO) mice, where SLAMF5 expression is completely abrogated, and performed transcriptional profiling. This allowed us to identify SLAMF5-regulated genes implicated in disease pathogenesis. As shown in the manuscript (Fig. 4E), SLAMF5 modulates the expression of several genes involved in myeloid activation and EAE pathology, including *Cd52*, *Irf7*, *Irf1*, *Ddx58*, *Itgax*, *Vcam1*, *Stat1*, *Bhlhe40*, and *Hspa1b*.

To validate the consistency of this SLAMF5-driven transcriptional program, we next assessed the expression of these genes—identified via RNA-seq from full KO mice—in additional models, SLAMF5 cKO mice (Fig. 5A) and N9 microglial cells (Fig. 5B), observing gene expression patterns consistent with those in the full KO model, thus confirming the robustness of the SLAMF5-regulated transcriptional network. Notably, we focused on *Cd52* due to its clinical relevance in multiple sclerosis (MS). A reduction in *Cd52* expression was consistently observed in SLAMF5 cKO mice (Fig. 5E–F), SLAMF5 antibody-treated mice (Fig. 5G–H), primary microglia (Fig. 5I–J), and N9 cells (Fig. 5K–L).

To further elucidate the mechanistic underpinnings, we investigated the regulation of *Cd52* by SLAMF5 through *Bhlhe40*, which was downregulated in both full KO and cKO cells. Our results demonstrate that SLAMF5 modulates the binding of BHLHE40 to the *Cd52* promoter region, providing direct evidence of a regulatory pathway.

Collectively, our data establish a mechanistic link between SLAMF5 and transcriptional control of genes critical for myeloid cell function and EAE progression. Therefore, our study goes beyond descriptive analysis and identifies a SLAMF5-driven pathway that governs immune cell behavior.

**The Cx3cr1-CreERT2 strain is widely used for multi-omic studies including the EAE model, one example here (Ennerfelt et al Cell 2022). The rationale to select global knockout strain over cKO approach "to ensure maximal and uniform deletion for transcriptomic analysis" should be addressed as a caveat in discussion.**

In this study, we employed both the full knockout (KO) and conditional knockout (cKO) models to investigate the role of SLAMF5. Genes identified by RNA sequencing in the full KO model were validated by RT-qPCR in the cKO mice (Figure 5A). Additionally, flow cytometry analysis (Figure 5E) demonstrated that *CD52* expression was downregulated in cKO mice, as well as in mice that received intracranial injections of a SLAMF5-blocking antibody. Thus, our use of both models was intentional and complementary—we did not favor the global KO over the cKO, but rather leveraged both to strengthen the validity and reproducibility of our findings.

**Histopathology: I commend the authors for their new experiment and inclusion of histological data from the control and cKO groups. The authors state "We performed spinal cord histology on EAE-induced cKO and control mice, which revealed significantly reduced lymphocyte infiltration in the cKO animals". Did the authors perform image quantification?**

The evaluation of histopathological findings was performed semi-quantitatively by an experienced pathologist, Dr. Ori Brenner. The assessment focused on grading the severity of lymphocytic infiltration in the spinal cord as mild, moderate, or severe. In control mice, lymphocytic infiltration was classified as severe across the cervical, thoracic, and sacral regions. In contrast, cKO mice exhibited only mild infiltration in the cervical and thoracic regions, with no detectable infiltration in the sacral spinal cord.
**Naive State Comparison: Statistics between naïve-control and Control EAE are not included in Fig 2K, M.**

Statistics is now included in the figures.

Reviewer comment 2:

**What role do the authors suggest microglial MHC-II plays in relation to SLAMF5 in EAE? It has been reported microglial MHCII is dispensable during EAE (Wolf et al Eur J Immunol 2018). Since the authors do not distinguish between microglia vs infiltrating myeloid cells in their analyses, how do the authors interpret the contribution of SLAMF5 in microglia vs peripheral myeloid cells?**

To distinguish between myeloid populations in the periphery and the central nervous system (CNS), we employed the conditional KO, *CX3CR1^CreER; SLAMF5^flox* mouse model, in which SLAMF5 expression is selectively downregulated in microglia and border-associated macrophages, while remaining unaffected in peripheral myeloid cells. Our results demonstrate that SLAMF5 expression in microglia and border-associated macrophages regulates disease progression and contributes to their activation, as evidenced by altered *MHCII* and *CD80* expression (Fig. 2). In contrast, SLAMF5 deficiency had no observable effect on myeloid cells in the spleen. These results were added to Supplementary Figure 5 and Figure 10.

Our results demonstrate that the absence of SLAMF5, in both full knockout and conditional knockout (cKO) mice, leads to reduced T cell infiltration into the central nervous system (CNS), accompanied by decreased IFN-γ secretion by these T cells (Fig. 1H–J and Fig. 2N–Q). These findings suggest that SLAMF5 expression on brain myeloid cells contributes to disease severity by promoting MHCII-mediated antigen presentation and subsequent T cell activation during EAE.

Reviewer comment 3:

**RNA-seq data**

**I still find the RNA-seq dataset difficult to interpret. There is 76% gene variance between 1 WT and the KOs, but it is unclear if this is because the authors sorted all CD45+CD11b+ cells from the inflamed CNS of EAE mice. The myeloid composition, and their activation states (microglial states, macrophages states, neutrophil states etc,) change dramatically from onset to peak disease (examples: Jordao et al Science 2019; Mendiola et al Nat Immunol 2020; Peruzzotti-Jametti et al Nature 2024). The selected genes in Fig. 5A support the reduced gene activation in CD45+CD11b+ cells of cKO brains in EAE.**

WT and SLAMF5 KO myeloid cells were sorted in parallel using a gating strategy for CD45⁺CD11b⁺ populations. Although there was some variability in disease scores among WT mice, the principal component analysis (PCA) plot shows clear separation of samples primarily along the x-axis (PC1), which correlates with disease severity and explains 76% of the total variance (Fig. 4A). The distinction between WT and KO groups is driven by a combination of principal components, rather than PC2 alone. While fully healthy animals were not included in the differential expression analysis, the strong internal consistency within the KO group and the stratification of WT samples by disease severity support the robustness of our findings.

We identified 395 significantly downregulated and 58 upregulated genes in SLAMF5 KO myeloid cells compared to all WT samples, underscoring the broad regulatory role of SLAMF5 during neuroinflammation. Differentially expressed genes (DEGs) were defined by an adjusted p-value < 0.05, an absolute fold change > 1.5, and a minimum read count of 30 in at least one sample.

Additional comments on revised manuscript

**1. The authors tend to present flow cytometric histograms of markers but then the quantification is a cell percentage. Is there a reason why the maker MFI is not presented? For example, but throughout manuscript, Fig 1E, F.**

MFI (Mean Fluorescence Intensity) indicates the average level of marker expression per cell, whereas the percentage of positive cells reflects how many cells express the marker. We used isotype controls to define the gating for activation markers such as MHCII and CD80, and analyzed the data using both approaches. Since the interpretation of results did not differ between methods, we chose to present only one for consistency.

**2. The title suggests a causal role of SLAMF5 in neuroinflammation in MS, which is too strong based on the presented data. A title reflecting MS models or CNS inflammation would be more appropriate.**

We chose to include "MS" in the title because, in addition to the animal models, our study incorporates analyses of myeloid cells derived from human peripheral blood and iPSC-based models—both of which are directly relevant to multiple sclerosis (Figure 7G–T). That said, we are open to revising the title to more clearly reflect the broader context of CNS inflammation and MS-related models if it would enhance clarity and alignment with the manuscript’s scope.

We believe that our clarifications included in the revised manuscript significantly strengthen our work and further support our conclusions. We respectfully request that you consider this revised version for publication in *PLOS Biology*.

Thank you for your time and consideration.

Sincerely,

Idit Shachar
