# Supplementary material for: The immune receptor SLAMF5 regulates myeloid-cell mediated neuroinflammation in multiple sclerosis
Source: PLoS Biol. 2025 Sep 8;23(9):e3003373. doi: 10.1371/journal.pbio.3003373 (PMC12431667; doi:10.1371/journal.pbio.3003373)
Supplement: Attachment — Submitted filename: Answers to the reviewers 13.8.docx [file pbio.3003373.s020.docx]

August 12^th^, 2025

Dear Melissa Vázquez Hernández,

Thank you for your letter dated July 13th regarding our manuscript, *“The immune receptor SLAMF5 Regulates Myeloid Cell-Mediated Neuroinflammation in Multiple Sclerosis”*, authored by Laura Bellassen, Keren David, Bar Lampert, Avital Sarusi-Portuguez, Michael Tsoory, Jazz Lubliner, Michael Osherov, Ori Brener, Ron Milo, Eran Hornstein, Shirly Becker-Herman, and myself. We are pleased that you are likely to accept this manuscript for publication, based on some revisions required. We revised the manuscript accordingly to address these points.

1. **Based on our Academic Editor's assessment of your revision, we are likely to accept this manuscript for publication. However, the Academic Editor requested that you please discuss the caveats of the full KO in the discussion. Please also make sure to address the following data and other policy-related requests.**

We addressed the caveats of the full knockout model in the Discussion section. We discussed the caveats of the full ko in the discussion section.

**2. Title**

The title was revised as suggested :” The immune receptor SLAMF5 regulates myeloid-cell mediated neuroinflammation in multiple sclerosis”.

**3. Please add the grant number in the Financial Disclosure statement in the manuscript details.**

This foundation does not assign a grant number.

4. **The Ethics statement needs to be a separate, independent (and the first) subheading in the Material & Methods section. It must include the full name of the IACUC/ethics committee that reviewed and approved the animal care and use, as well as the protocol/permit/project license number.**

We added the IACUC/ethics committee name and number to the Material and Methods section.

5. **You may be aware of the PLOS Data Policy, which requires that all data be made available without restriction: http://journals.plos.org/plosbiology/s/data-availability. For more information, please also see this editorial: http://dx.doi.org/10.1371/journal.pbio.1001797**

**Please supply the numerical values either in the a supplementary file or as a permanent DOI’d deposition for the following figures:**

**Figure 1BDF-J, 2CEGHIKM-Q, 3ABDFGHJ-NPR, 4A-E, 5ABCEGIK, 6BDFHJK, 7GIKMOQS, S1AB, S2ABC, S3AB, S4BC, S5AB, S6AB, S7, S8AB, S9, S10, S11**

**NOTE: the numerical data provided should include all replicates AND the way in which the plotted mean and errors were derived (it should not present only the mean/average values).**

All the information is now included in the Excel file *S1 Data*.

**6) For figures containing FACS data (Figures 1ACE, 2BDFJL, 3CEGIOQ, 5DFHJL, 6ACEGI, 7A-FHJLNPRT, S4A), please provide the FCS files and a picture showing the successive plots and gates that were applied to the FCS files to generate the figure. We ask that you please deposit this data in the FlowRepository (https://flowrepository.org/) and provide the accession number/URL of the deposition in the Data Availability Statement in the online submission form.**

There appears to be an issue with the website—we’re receiving the following notification: “We have temporarily disabled the creation of new experiments as we are continuously running out of space.”

**6. Supplementary files (e.g., excel). Please ensure that all data files are uploaded as 'Supporting Information' and are invariably referred to (in the manuscript, figure legends, and the Description field when uploading your files) using the following format verbatim: S1 Data, S2 Data, etc. Multiple panels of a single or even several figures can be included as multiple sheets in one excel file that is saved using exactly the following convention: S1_Data.xlsx (using an underscore).**

Supplementary files were uploaded in the right format and the name is S2_Data.pdf

**7. Please add a scale bar in the following microscopy pictures in Figure 2N.**

Scale bar was added to the figure.

**8. You mentioned the Data was submitted to GEO. However, it is not clear which data was submitted there. Please also provide the accession code or a reviewer link so that we may view your data before publication. Please ensure that your Data Statement in the submission system accurately describes where your data can be found and is in final format, as it will be published as written there. I would also like to point out that in your text, there is no statement of Data Availability, please provide it.**

The data have been submitted and will be made publicly available upon acceptance of the manuscript. We will include the GEO accession number as soon as it is assigned.

**9. Per journal policy, if you have generated any custom code during the course of this investigation, please make it available without restrictions. Please ensure that the code is sufficiently well documented and reusable, and that your Data Statement in the Editorial Manager submission system accurately describes where your code can be found.**

We did not generate any custom code.

**10. Please provide separate figure files in .tif or .eps format. For more information about figure files, please see our guidelines**

Tiff files were uploaded.

**11. We do not publish any copyright or trademark symbols that usually accompany proprietary names, eg ©, ®, ™ (e.g. next to drug or reagent names). Please remove all instances of trademark/copyright symbols throughout the text, including:**

® on page 9.  ™ on page 7.

The symbols were removed

**12. We have noticed that you have uploaded Supporting Information files, but you have not included a list of legends. Please add a full list of legends for your Supporting Information files after the references list.**

Legends were added and the name is S3_Data.pdf

We believe that our clarifications included in the revised manuscript significantly strengthen our work and further support our conclusions. We respectfully request that you consider this revised version for publication in *PLOS Biology*.

Thank you for your time and consideration.

Sincerely,

Idit Shachar
